# *“They just want to stop feeling what they’re feeling”*: A qualitative investigation and characterization of coping strategies for mental distress among adolescents in American Samoa

**DOI:** 10.1371/journal.pmen.0000669

**Published:** 2026-08-10

**Authors:** Julia Winschel, Emma J. Mew, Vanessa Blas, Leiema Hunt, Si’itia Soliai-Lemusu, Sarah R. Lowe, Joshua Naseri, Robert L. M. Toelupe, Nicola L. Hawley, Jueta McCutchan-Tofaeono

**Affiliations:** 1 Department of Social and Behavioral Sciences, Yale School of Public Health, Yale University, New Haven, Connecticut, United States of America; 2 Warren Alpert Medical School of Brown University, Providence, Rhode Island, United States of America; 3 Department of Chronic Disease Epidemiology, Yale School of Public Health, Yale University, New Haven, Connecticut, United States of America; 4 Duke Global Health Institute, Duke University, Durham, North Carolina, United States of America; 5 Center for Global Mental Health, Duke University, Durham, North Carolina, United States of America; 6 Department of Health, American Samoa Government, Pago Pago, American Samoa, United States of America; 7 Obesity, Lifestyle, And Genetic Adaptations (OLAGA) Study Group, Pago Pago, American Samoa, United States of America; 8 Malama Navigator Counseling & Consulting, Pago Pago, American Samoa, United States of America; 9 Empowering Pacific Island Communities (EPIC) Pago Pago, American Samoa, United States of America; PLOS: Public Library of Science, UNITED KINGDOM OF GREAT BRITAIN AND NORTHERN IRELAND

## Abstract

Adolescents in American Samoa face a high mental health burden, with many youth experiencing symptoms of depression, anxiety, substance use, and suicidality. Understanding the strategies that adolescents use to cope with stressors can help strengthen existing mental health promotion and psychoeducational resources. We aimed to identify common coping strategies used by adolescents in American Samoa in response to mental distress. Using a two-stage qualitative approach, semi-structured interviews were conducted with a diverse sample of adult community members (*N* = 28) from American Samoa. Later, five focus groups with adolescent participants (*N* = 35, aged 13–18 years) were conducted to validate themes identified among adult key informants. Transcripts were coded in duplicate and analyzed using thematic analysis. Coping strategies were characterized as approach or avoidant according to Billings and Moos’ (1981) existing framework. Key informants and adolescents identified coping strategies that fell into three major domains: (1) Avoidant coping strategies aimed to divert attention away from the stressor or suppress distress and were sometimes associated with high-risk responses, including self-harm, suicide, and substance use; (2) Approach coping strategies were generally supportive and involved engaging with distress, seeking connection, or expressing emotions, including through social engagement and confiding in peers; and (3) Mixed coping strategies that had characteristics of avoidant and approach depending on the context in which they were used (prayer, social media). Utilizing a collaborative and community-partnered approach, these findings provide a foundation to develop mental health promotion and psychoeducational initiatives to help adolescents better understand their own stress and build healthy coping strategies.

## Introduction

American Samoa is an unincorporated US territory in the South Pacific. Though many youth are thriving in this context, recent studies have noted a high prevalence of mental health problems among adolescents [1,2]. In 2021, a community-led survey of *N =* 1,125 high school students in American Samoa found that almost 30% of adolescents had made a suicide attempt in the past year [1]. Qualitatively, Samoan adolescents report dealing with a number of stressors, ranging from school-related pressure to family conflict and challenges navigating their cultural identity [3]. In many other settings, coping skills have been explored as predictors of mental health outcomes, including the risk of suicidal ideation and the likelihood of a fatal suicide attempt. To date, the behaviors used by Samoan youth to manage their stress have been unexplored. An understanding of how adolescents cope would be especially important in this setting; with limited mental health treatment available [2], there is a need for feasible and scalable mental health promotion initiatives to support adolescents in coping with life stressors.

A longstanding body of research has both theorized and empirically demonstrated that coping styles tend to fall along two dimensions: approach and avoidance, each defined by cognitive and emotional activities that are oriented either toward - or away from - a given threat, respectively [4–6]. Approach coping includes strategies that aim to understand and ultimately resolve the threat, such as information gathering and problem solving [6]. In contrast, avoidance coping includes strategies that foster cognitive and emotional distance from a stressor, including distraction, denial, and emotion-focused coping. Although approach and avoidance coping can each be advantageous depending on the potential to control a given situation, there is evidence that individuals who consistently use avoidance coping are at risk of a range of negative behavioral outcomes, including suicidality, self-harm, and substance misuse [7–9]. Much of the existing research that applies this model to adolescent coping in other settings utilizes quantitative methods. For instance, studies have examined associations between coping strategies and psychopathology among adolescents [10], the role of gender, social support, and age in coping [11], and the relationships between coping styles, peer victimization, and health outcomes [12]. While quantitative methods have allowed researchers to measure the prevalence of general coping styles among adolescent samples and identify which coping behaviors function within specific contexts, they limit the depth of understanding gained from individual perspectives. In contrast, qualitative research designs allow adolescents to provide specific insight into how they prefer to manage common stressors. Further, while the two-dimensional approach versus avoidance model of coping has been previously applied to samples of adolescents in Western contexts, it has not to our knowledge been studied in the context of Samoan or Polynesian communities.

In Western societies, individualistic approaches to coping, such as managing challenges alone, with private emotional outlets, or individualized counseling are commonly used [13]. However, in cultures rooted in collectivism, approaches to coping generally rely more heavily on social and community support systems, as the self is viewed as interconnected with other [14,15]. Among Polynesian communities, cultural values based on pluralism, communalism, and associated religious and spiritual beliefs have been shown to influence strategies to cope with mental health challenges [16]. In the only study, to our knowledge, to examine coping strategies specifically, Allen and Heppner examined religiosity, collectivist coping, and psychological well-being among *N* = 94 Polynesian adults residing in the Midwestern US and found, for example, that those who were committed to their religious faith were more likely to use collectivist coping styles, including relying on family support [17]. Further, Allen and Smith examined associations between collectivist coping styles and mental distress among Polynesian Americans and found that the reliance on family support among this group was significantly more predictive of positive psychological well-being compared to the use of private emotional outlets [18].

More recent work with Pacific and Pasifika adolescents underscores the importance of stigma, confidentiality, family expectations, cultural identity, and culturally responsive support in shaping mental health help-seeking. For example, Garrett et al. [19] found that Pacific Islander male adolescents described stigma, consequences of disclosure, and cultural misalignment as barriers to seeking mental health support. Similarly, qualitative work with Pasifika young people in South Western Sydney identified shame, trust, confidentiality, and family and community context as important influences on help-seeking for mental health and substance use concerns [20]. Although this literature focuses more on help-seeking than coping strategies specifically, it supports the need to interpret adolescent coping in Pacific communities within relational, cultural, and community contexts.

Using data collected as part of a larger community-partnered qualitative project investigating mental health among adolescents in American Samoa [2], the current study aimed to identify common coping strategies used by American Samoan youth in response to emotional stress and to characterize using an approach/avoidance framework. Because this framework was developed largely in Western psychological research, we applied it as an organizational tool while considering the relational, spiritual, and community contexts in which coping occurs in American Samoa. Exploring common and accessible coping strategies among youth may provide local policymakers and stakeholders with information needed to advance psychoeducational programs to support youth mental health.

## Methods

### Ethics statement

This study was reviewed and approved by the Yale University (#2000028354) and the American Samoa Department of Health (#00001249) Institutional Review Boards. All participants provided their written informed consent/assent for participation; parental consent was also obtained for adolescent participation. Referral procedures were in place for any disclosure of suicidal ideation or conditions that would place the adolescent in immediate harm (such as an unsafe home environment) during discussions with clinicians among the team available to offer immediate assessment and appropriate action.

### Study design

This work was part of a larger qualitative project that aimed to understand adolescent mental health in American Samoa more broadly, using open, non-directive questions that focused on defining mental health and mental illness, exploring common mental health problems, barriers and facilitators to mental health care, existing coping strategies, and potential interventions to improve the current mental health prevention and treatment infrastructure. Further methodological information, including interview guides and codebooks, has been published previously [2,3,21].

Briefly, the study design, analysis, interpretation, and dissemination used a community-partnered model. Guided by the Samoan *fa’afaletui* framework, which acknowledges the importance of weaving perspectives together to reach consensus [22,23] we sought to include diverse perspectives from ‘the top of the mountain’ (policy makers, non-governmental organizations, and mental health providers who could appreciate the broader scope and scale of the challenges with adolescent mental health), ‘the top of the tree’ (adults who work directly with adolescents), and ‘the person in the canoe fishing’ (those most impacted, such as adolescents and young adults) [22].

In a two-stage qualitative approach, we conducted in-depth, semi-structured interviews with *N* = 28 adult (>=18 years) key informants (KIs) who lived in American Samoa between October 19 2020 and February 7 2021 and then focus groups with adolescents [2,3]. We used a two-stage approach intentionally so that the research team could first learn from experts about the mental health landscape in American Samoa, the resources available to youth, and common mental health challenges. This allowed us to approach adolescent focus groups with sufficient knowledge to probe around resource utilization, and coping strategies, among other topics. Eligible KIs needed to have familiarity with adolescent mental health, speak English, and represented one of the perspectives described above. We initially approached our partners at the Department of Health and then used snowball sampling to recruit additional participants, intentionally seeking diversity in job roles and perspectives. To further explore and validate themes arising from the semi-structured interviews, we conducted five focus groups with adolescent participants (*N* = 35; aged 13–18 years, average age = 15.8 years) [3]. Recruited through personal networks, snowball sampling, and social media advertising, adolescents were eligible if they were English-speaking, identified as Samoan and were enrolled in middle- or high school; although all participants who ultimately enrolled were attending high school at the time of participation. All semi-structured interviews with adult KIs and adolescent focus groups were conducted in English over Zoom or by phone. Focus groups were facilitated by two research team members [JN, LH] with training in focus group facilitation and experience working with youth in American Samoa. Between May 6 and June 4, 2022, adolescents participated in each group and discussions lasted between 2.25 and 2.75 hours. For both interviews and focus groups, a semi-structured agenda was used (published previously [3]) with broad prompts about drivers of poor mental health leading to more in depth discussion of available treatment, coping strategies, and needs for further support. In focus groups, dotmocracy exercises were used to validate themes from adult interviews. Saturation was assessed during each qualitative data collection stage through interview and focus group debriefs by the study principal investigator and team discussion. Because coping strategies were one component of a broader qualitative study on adolescent mental health in American Samoa, saturation was assessed globally across the major domains of the parent study, including understandings of mental health and mental illness, common mental health concerns, barriers and facilitators to care, coping strategies, and potential interventions. Saturation was indicated when additional interviews or focus groups no longer produced new substantive themes across these broader domains. For the current analysis, coping-related content was sufficiently recurrent and elaborated across both adult key informant interviews and adolescent focus groups to support a focused analysis of coping strategies.

### Coding and thematic analysis

A list of initial codes was developed using deductive thematic analysis that focused on semantic themes [24]. Members of the research team pilot tested the initial codes on a sample of transcripts from adult KI interviews to iteratively develop the codebook. The final codebook was used to analyze all semi-structured interviews with adult KIs and was later used to analyze youth focus group transcripts following the addition of several codes based on further pilot testing. All transcripts were coded in duplicate; study principal investigator EM coded all transcripts. Coding pairs and members of the analytic team met regularly to discuss differences in interpretation, refine code definitions, and document analytic decisions. We did not calculate inter-coder agreement because our goal was not to establish a single objective coding solution, but to use duplicate coding as a tool for reflexive discussion and analytic depth. When coding differences reflected clear misunderstanding or inconsistent application of the codebook, the team clarified the code definition and revised coding as appropriate. When multiple interpretations were supported by the data, these interpretations were discussed and retained rather than forced into a single consensus interpretation.

After thematic coding was complete, the first author developed an initial categorization of identified coping strategies using the approach/avoidance framework. These categorizations were reviewed by the full author team, including Samoan team members and collaborators involved in the design, data collection, analysis, and interpretation of the broader study. Strategies were categorized according to their described function in participant narratives: whether they appeared to help youth engage with distress, seek support, express emotion, restore connection, or address a stressor (approach); whether they appeared to help youth disengage from, suppress, conceal, or escape distress (avoidant); or whether their function varied substantially by context (mixed). The mixed category was not determined *a priori* or used to indicate disagreement among authors; rather, it was included to reflect participants’ own descriptions of prayer and social media as strategies that could function differently depending on context, intention, and response from others. Illustrative quotes from both adult KIs and youth focus group participants are included to demonstrate the variety and perceived effectiveness of specific coping strategies.

### Reflexivity statement

The research team included investigators and community partners with varied relationships to American Samoa, including Samoan team members, collaborators based in American Samoa, clinicians and community leaders with experience in adolescent mental health, and university-based researchers from outside American Samoa. The first author and several US-based co-authors approached this work as outside researchers and recognize that their disciplinary training, institutional location, and cultural positionality shaped data interpretation. At the same time, the senior US-based author has worked collaboratively with partners in American Samoa for more than 15 years, providing long-standing contextual knowledge and established community relationships. Throughout analysis, team discussions were used to examine how professional background, cultural position, and proximity to the community shaped interpretation of participant narratives.

## Results

KI perspectives (*N =* 28) came from ‘the top of the mountain’ (*N=*15; mental health professionals), the top of the tree’ (*N =* 9; educators, NGO representatives, service administrators, other community stakeholders), and ‘the person in the canoe fishing’ (*N =* 4; young adults). Adolescent focus groups (*N =* 35 total participants) included *N =* 19 girls, 15 boys, and one individual who identified themselves as non-binary. The mean age of adolescents was 15.8 years (13–18 years), all were currently attending high school, and 91% identified as Samoan (others reported mixed ancestry). To attribute quotes to KIs or adolescents we use the following labeling system: adult KIs are referred to as “A” and focus group participants are referred to as “Y” (youth). For KIs, the superscript identifies perspectives from the top of the mountain (“M”), top of the tree (“T”), or person in the canoe fishing (“C”); for adolescents, the superscript identifies their gender as male (“M”) or female (“F”).

Because the study used a two-stage qualitative design, the results below distinguish between adult key informants’ perceptions of adolescent coping and adolescents’ own descriptions of coping where possible. Adult key informants often described coping from the perspective of clinicians, educators, service providers, and community members who observe youth distress and its visible consequences. Adolescents more often described the internal experience of coping, including the desire for control, privacy, emotional relief, connection, and nonjudgmental support. Across themes, we note where adult and adolescent accounts converged and where a strategy was primarily described by one group. Overall, adult and adolescent accounts converged around the importance of peer support, creative expression, prayer, and social media, while adult participants more frequently emphasized substance use, aggression, and other externally visible high-risk behaviors.

Through analysis, we interpreted participants’ descriptions of coping as falling along an avoidance-approach spectrum, with some strategies occupying a context-dependent middle ground; as such we organized responses into three domains/themes. Participants described avoidance-oriented responses as those that helped adolescents escape, suppress, internalize, or gain distance from a stressor or the emotions associated with it. In contrast, approach-oriented strategies involved efforts to engage with distress more directly, including through emotional expression, support-seeking, connection, meaningful activity, or problem-solving. Interestingly, some coping strategies could be both avoidant and approach (mixed) depending on context, such that they are placed in the middle of the spectrum. In general, adult and adolescent perspectives on coping were congruent; instances where perspectives differed are noted below.

**Fig 1:**
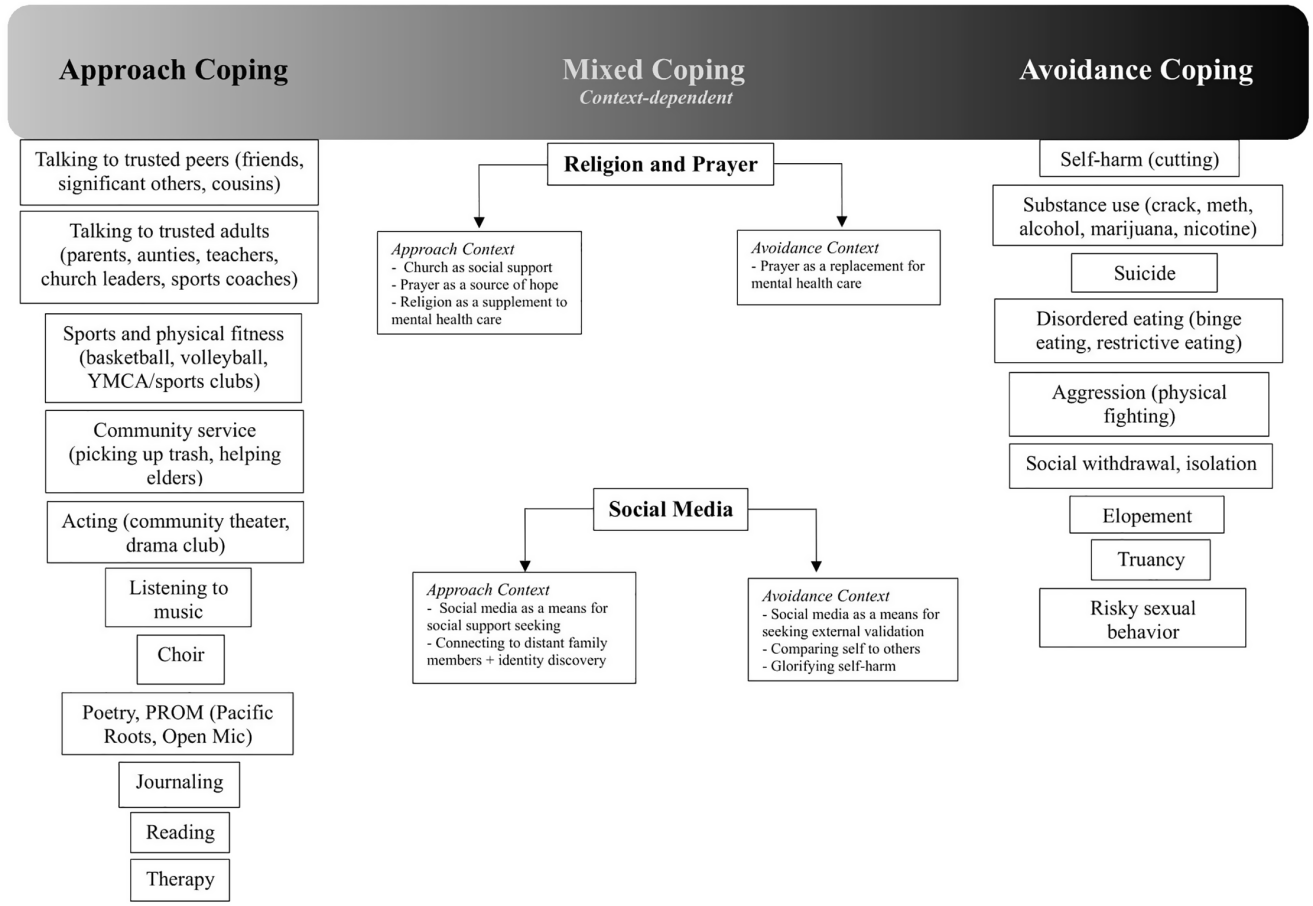
Specific coping strategies described by participants, organized along the analytic approach–avoidance coping spectrum. Within each category, strategies are arranged vertically according to relative code frequency in the qualitative data, with more frequently coded strategies presented toward the top of the diagram and less frequently coded strategies toward the bottom. This ordering is intended to reflect relative salience within the coded interview and focus group data and should not be interpreted as an estimate of population-level prevalence. Mixed coping strategies, including religion/prayer and social media, are further subdivided based on characteristics described by participants as consistent with approach-oriented or avoidance-oriented coping.

Fig 1 summarizes the full range of coping strategies described across interviews and focus groups. Strategies presented in the figure are ordered within each domain according to relative code frequency, with more frequently coded strategies appearing higher in each column. Because this was a qualitative study, this ordering is intended to indicate relative salience within the coded data rather than population-level prevalence. The results below focus on the strategies that were most elaborated by participants, while Fig 1 also includes less frequently discussed strategies that were mentioned by a smaller number of participants.

### Theme 1: Avoidance coping: “They just want…to stop feeling what they’re feeling” (A-ID#05^C^)

Adult key informants most often described avoidance-oriented coping in terms of visible or high-risk responses to distress, including substance use, aggression, self-harm, and suicidal behavior. Adolescents also directly discussed self-harm, particularly as a response to feeling overwhelmed, trapped, or unable to control other sources of distress. Across both groups, these behaviors were described as ways that some youth attempted to numb, escape, suppress, or gain temporary relief from emotional pain.

Participants stated that youth use coping mechanisms that allow them to distract, numb, or hide from their problems. In this way, adolescents internalize their emotions, precipitating negative behaviors directed towards themselves. Self-harm, suicide, and use of alcohol and drugs (specifically crack, meth, marijuana, and nicotine) were perceived to be among the most visible high-risk responses associated with avoidance-oriented distress:

*“I know a lot of people, they kind of get stuck in their own head…and feel like they’re drowning in their own thoughts. I think that would be one reason why they would turn to meth…they want to get high; they want to forget everything*.” (A-ID#02^C^)

Youth also commonly described self-harm as a mechanism for coping with perceived lack of control in their lives:

*“In some cases [self-harm] is sort of a form of control…we don’t really have control over the other factors that other people play into our mental health, but we kind of have control over…how much pain we inflicted on ourselves through cutting.*” (Y-ID#13^F^)

*“Probably…having so much pressure from school or…relationships or…things like that…[Youth] feel like they can’t live up to that standard. So then they, they just turned to something where they feel like they can’t talk about it. So they [self-harm]...sort of as a coping mechanism.”* (Y-ID#43^F^)

Participants also described several less frequently discussed avoidance-oriented or high-risk responses to distress, including disordered eating, social withdrawal or isolation, truancy, risky sexual behavior, and elopement, which we use here to refer to running away from or leaving home, school, or another supervised setting without permission. These behaviors were not described in the same depth as self-harm, suicidal behavior, or substance use, but they were included in Fig 1 because participants identified them as ways that some adolescents attempted to escape, conceal, or manage overwhelming distress.

A minority of adult KIs also stated that youth use coping mechanisms that involve outward displacement of emotions targeting others. Specifically, physical fighting and aggression were identified as behavioral manifestations of youth’s avoidance of negative emotions:

*“…They end up fighting…just because of pent up anger, frustration from situations at home then carries it on to the school and [are]…looking for a fight…or a reason to relieve or be able to take out their anger on somebody else*.” (A-ID#21^M^)

### Theme 2: Approach coping: “It’s kind of like turning a negative into a positive” (Y-ID#06^M^)

Approach-oriented strategies were described by both adult key informants and adolescents, but adolescents provided particularly detailed accounts of strategies that helped them feel connected, understood, grounded, or able to express distress without shame. Participants described common coping mechanisms that allow youth to manage their emotions using beneficial behaviors that directly or indirectly addressed their stressors. Such behaviors provided a sense of wellbeing to offset negative emotions associated with stressors and shifted focus away from the stressor itself. Though not directly managing distressing emotions, these coping strategies were described as decreasing the amount of space that a stressor took up in adolescents’ minds. Specifically, participants frequently recognized community or social engagement as a common coping strategy amongst youth, giving them a sense of worth, purpose, and control over their lives:

“…*being connected to youth groups, to other service organizations, so that it helps them to see something beyond what’s going on in their own lives or to help them feel a sense of control or feel a sense…that they’re contributing to something in different ways, and that sense of belonging.*” (A-ID#10^M^)

Youth participants frequently agreed that pursuing a passion, hobby, or relationship to ground them was important for maintaining a sense of identity and improving mental health:

“*I would have to say the difference between youth who experience passive suicidal ideation and those who actively try to harm themselves and commit suicide would be…having something…that they’re really passionate about or that they have love for, this could be music or one of their friends or family members, or for some even God or another form of religion. It’s just something sort of like the light…you see amidst the darkness*.” (Y-ID#13^F^)

Participants also explained that youth use coping strategies that involve addressing their negative emotions directly to better understand or manage them. Specifically, participants mentioned communicating openly about stressors with trusted peers as a means for finding a sense of comradery while decreasing feelings of loneliness and shame, although they described this being a more common practice among girls:

*“[Talking to friends] helps in a way…you tell someone about your experience and then they’ll mention…a similar experience, which could help because [they realize they’re] not the only person that has gone through this…or I’m not the only person that feels this way.*” (A-ID#05^C^)

“*…Girls, we have a very active community and we help each other…and we can talk about [mental health], but for boys, I feel like it’s not [an] open conversation where they can go and…to one another and say, Hey, I need to talk about this…I feel like it’s harder for [boys] to open up about [mental health] because it’s not that accepted for them.”* (Y-ID#33^F^)

Although participants most often emphasized peers as trusted confidants, they also described some adolescents turning to trusted adults, including parents, aunties, teachers, church leaders, and sports coaches, when those adults were perceived as safe, nonjudgmental, and able to provide guidance.

Adult KIs identified therapy as an alternative, though infrequently utilized, environment for discussing emotions openly, suggesting “...*therapy is a place where we process…you’re able to share your thoughts, your opinions, your emotions, and…being able to do that helps to purge some of those negative emotions*” (A-ID#15^M^). Youth participants suggested that even in the absence of a trusted family member or friend with whom they could discuss their emotions, using journaling, “*writing down your problems, communicating with yourself and writing down all the stuff that you [want to] but can’t open up to somebody else [about]”* (Y-ID#11^F^) as an important strategy for working through problems without bottling them up.

Participation in theater or other programs that encourage creative expression was commonly cited by adults as a way for youth to explore emotions from different perspectives and to have a platform to share their stories while listening to others, creating a sense of community and belonging:

“*I have a friend who… really believes that theater can be a powerful means of healing…[theater] gives kids an opportunity to express themselves through acting and even express and participate and be a part of a team through creating set designs*.” (A-ID#09^T^)

Pacific Roots, Open Mic (PROM) events for youth in American Samoa were particularly noted as providing a safe space for adolescents to express their thoughts and emotions creatively via performance arts. One adult KI stated, “…*Pacific Roots, Open Mic is not new. It’s been around since 2015…we’ve seen people use this platform as a coping mechanism…just talking out…hearing stories, poems, just having an open space for them, a free space for them to express themselves…As far as participating, being part of just listening to it, it gives them courage and…the courage to actually speak their truth*” (A-ID#25^T^). Youth participants agreed that PROM provides beneficial support for youth. One adolescent stated, “*The reason why…[PROM] helps to solve our mental health…[because] most of the PROM, we all have partners. So we know that someone is there for [us] and there’s someone [who] cares for me. That’s why I think it’s helpful.”* (Y-ID#8^F^). Another youth participant added, “*[PROM] will help others to come out of their comfort zone of having the ability to share what they’ve gone through.”* (Y-ID#07^M^). Such programs encourage youth to express their emotions more publicly, thus increasing their sense of strength, allowing for the development of empathy for their peers, and helping youth work through emotional challenges and explore solutions for their problems. Other approach-oriented strategies, including listening to music, choir, reading, sports, and physical activity, were described as helping adolescents regulate emotion and maintain a sense of identity.

### Theme 3: Mixed approach-avoidance coping

Adult and adolescent participants both described prayer and social media as common coping strategies, but their accounts emphasized the importance of context. Adult key informants were more likely to identify circumstances in which these strategies could delay disclosure, substitute for formal support, or expose youth to harm, whereas both adults and adolescents emphasized their potential to provide comfort, connection, expression, and validation. Taken together, these accounts led us to characterize prayer and social media as mixed strategies. Specifically, prayer and social media were described as having the potential to fall within the avoidant domain if they are used as an escape from the stressor, and the approach domain if they are used as platforms for emotional exploration and support seeking.

Participants explained that the use of prayer as a coping strategy is variably helpful, depending on the extent to which prayer is used for coping and the purpose that adolescents deem prayer to serve. Though participants stressed the importance of prayer and connection to spirituality as a protective factor for youth mental health, some adult KIs also suggested that prayer can be reductionist in the sense that it can minimize attempts to understand and address underlying causes of mental health challenges:

“*The people that were facilitating [the suicide prevention summit] were just church members and…the whole message was if you’re depressed, just turn to God, which I thought was kind of inappropriate because…people can have chemical imbalances in their brain, which caused them to be depressed, or…there [are] other factors. And sometimes turning to God just doesn’t fix it. People need actual help*.” (A-ID#05^C^)

Participants also explained that prayer can be used as a replacement for talking about stressors, making it a mechanism for adolescents to bottle up their emotions:

“*I think that we…make [adolescents] feel like they should pray instead of talking to somebody*.” (A-ID#05^C^)

While participants did not describe prayer as inherently harmful, they noted that its benefits may be limited when prayer and formal help-seeking are treated as mutually exclusive. In other interviews, adult KIs identified involvement with the church as “*a very strong source of comfort or reassurance or spiritual care*.” (A-ID#01^M^):

“*I know a lot of kids here…in light of the recent suicides have really been trying to turn to God and everything, to…help them through whatever they’re going through…they view God as…the light at the end of the dark tunnel for them*.” (A-ID#02^C^)

Youth participants largely agreed that prayer is an important and common coping mechanism and stressed that formal help seeking and prayer do not have to be mutually exclusive.

Social media was described as having both helpful and harmful implications depending on the underlying intention of use and the function that adolescents believe social media to have. Adult KIs suggested that social media can be used to present a façade, thus providing a platform to avoid acknowledging negative emotions and stressors:

“*I think with social media too, we like to show that we’re happy and we’re ok. And [that] we have friends and that everything is going good for us*.” (A-ID#05^C^)

More positively, participants also identified social media as a common means for emotional exploration and seeking social support from peers about their problems:

“*Social media [is] just an opportunity for people to be able to…bring out some of these things that they’re [experiencing], and they’re figuring out how to communicate it, to verbalize it…So in a culture where you’re not taught to do that, and then now there’s an opportunity to do that. And also where you’re able to do that and also not…be reprimanded… that’s the thing with social media, you can create a whole different persona…but I think it’s just an avenue that allows [adolescents] to go through some of that emotional process…Of being able to verbalize…some of the emotions that they’re experiencing*.” (A-ID#15^M^)

While the sharing of personal experiences and emotions surrounding them was thought to benefit adolescents who feel they lack a trusted adult or peer with whom they can discuss their stressors offline, many participants also suggested the outcome of using social media as a coping strategy is dependent on both what one chooses to post and the audience that the message reaches. One adult KI explained how social media can harm mental health if vulnerability is not met with validation or action:

*“…the most recent suicide, the kid even posted it on Facebook, that [he was] gonna kill [himself]. And he said how he’s going to do it…And nobody responded to that. Nobody said, you know, are you okay? Let’s talk or let’s meet…he posted it on Facebook and nobody responded*.” (A-KI#24^T^)

Youth participants agreed that if a social media audience has poor intentions, social media can harm mental health:

*“…social media can be a way of venting or expressing your feelings. But at the same time, others can use that against you and what you put out there and…there are influences on social media that could keep you even more depressed*.” (Y-ID#29^F^)

Aside from how others respond to youths’ posts on social media, participants explained that simply exploring others’ lives on social media and being constantly inundated with posts from peers can have consequences for adolescent mental health. Particularly, participants reported that social media encourages adolescents to compare themselves to others and use social media engagement as a proxy for social status:

*“…social media [is] a major factor in having bad mental health…first, a lot of people like to compare themselves to others on social media. Second…using [social media] is a good way for [adolescents] to vent…but then at the same time, social media can be really draining…because we’re constantly trying to check out social media, see if someone liked our post, see if anyone commented under our post*.” (Y-ID#29^F^)

Further, some commented that social media use has the potential to exacerbate feelings of isolation and disappointment:

“*And you might be on social media, seeing what you’re missing out on, seeing how much better life could be in someone else’s home or somewhere in the States. I think social media has done good in connecting families that are separated. It has also become a platform to really emphasize what you’re missing in your own life*.” (A-ID#03^T^)

## Discussion

To our knowledge, this study is the first to attempt to understand coping strategies used by American Samoan youth in response to emotional stress. KIs and focus group participants reported that adolescents use a variety of coping strategies that can be characterized along an avoidance-approach spectrum. Importantly though, in a setting where social norms, religious beliefs, and family and peer relationships strongly shape the coping methods used by youth in response to distress, the function of any coping strategy depends heavily on context.

We selected the approach/avoidance model because it provided a useful organizational framework for characterizing diverse coping strategies according to their perceived function. The framework helped distinguish strategies that participants described as moving adolescents toward emotional expression, support, connection, or meaning-making from those described as helping youth suppress, conceal, numb, or escape distress. However, applying this model in American Samoa requires attention to culturally specific meanings and contexts. Because the model was developed largely in Western psychological research [4–6], it does not fully capture the relational, spiritual, and community-based dimensions of coping in American Samoa, where wellbeing is closely tied to family, church, village, and peer relationships. In this context, strategies that may appear to be distraction in an individualistic framework, such as music, sports, community service, youth groups, or prayer, may also function as relational and meaning-making practices. Similarly, the classification of self-harm, suicidal behavior, and substance use as avoidance-oriented requires careful interpretation. We do not conceptualize these behaviors as coping strategies equivalent to help-seeking, journaling, prayer, or other everyday responses to stress. Rather, participants described them as harmful, crisis-related, or high-risk responses to distress that may serve an escape-oriented function when adolescents feel unable to disclose, regulate, or resolve overwhelming emotional pain. This distinction is important because interpreting these behaviors functionally should not normalize them or minimize their severity, but instead highlight the need for prevention, timely clinical response, and safer community-based pathways to support.

Understanding how adolescents cope in American Samoa requires attention to the relational contexts in which distress is experienced, interpreted, and responded to. Coping is not only an individual response to stress; it is shaped by family expectations, peer relationships, church and village life, and concerns about privacy, shame, and belonging. These relational contexts influence whether a strategy creates opportunities for support, disclosure, and connection, or instead reinforces silence, stigma, or withdrawal. Although there is no prior published research from American Samoa specifically, researchers have asserted that cultural and social values, including religion and communalism, shape coping strategies and health-seeking behaviors among other Polynesian populations [18,25]. American Samoan cultural norms are founded on values of mutual respect, trust, and traditional social roles that support the community as a whole [1]. Polynesian cultures view the family as the central source of coping with emotional challenges [17], to the extent that seeking help outside of the family is sometimes considered shameful – for both the individual and family. At the same time, in American Samoa, families are a known source of both stress [3] and interpersonal mental health stigma via shaming and dismissal of emotion [21], resulting in youth feeling stuck between the expectation to rely on their families for support and the exacerbation of stress that results from family interactions or stigma at home. Specifically, adolescents may avoid addressing their emotional needs with parents due to pressure to fulfil familial expectations, not wanting to disappoint family members, and for fear of judgement from the broader community [3,17]. Any attempt to intervene at the family level to support youth mental health would need to be cognizant of this complexity, attempting to strengthen existing relationships while providing the problem-solving skills to overcome family-induced stress.

Although family support is a foundation of collectivist culture, participants mentioned support from family members relatively less frequently than they did support from peers as an important coping strategy among American Samoan youth. Although this is in line with recent qualitative work highlighting the importance of peer bonding as a source of emotional support among New-Zealand-born Samoan youth [26], this pattern should not be interpreted as evidence that family is unimportant. Rather, it may reflect several overlapping processes. Developmentally, adolescents may turn to peers as they seek privacy, autonomy, and validation from others who they perceive as sharing similar experiences. Culturally, however, family remains central to identity, obligation, and belonging. Participants’ accounts suggest that family may occupy a dual role: it can be a foundational source of support, but it can also be a source of stress, shame, or dismissal when adolescents disclose emotional distress. This complexity may leave some youth feeling caught between the expectation to rely on family and the fear that disclosure will produce judgment or disappointment, a tension also described in recent work on Pacific Islander adolescents’ mental health help-seeking [19,20]. Methodologically, because the broader interview and focus group guides focused on adolescent mental health, stress, service barriers, and coping, participants may also have emphasized peers as more immediate or accessible confidants in the context of emotional distress. Taken together, these findings suggest that interventions should not replace family-centered approaches with peer-focused approaches but should consider how peer and family support can be strengthened simultaneously.

Unsurprisingly, seeking individualized mental health treatment was seldom identified by participants as a coping strategy used among youth. Mew et al. identified several barriers to adolescent mental health care in American Samoa, including a lack of mental health literacy, concerns about confidentiality, and a severely limited local mental health workforce [2]. Such barriers greatly restrict the accessibility of individual therapy for youth facing mental health problems, highlighting the importance of interventions that improve mental health support within families and peer groups in American Samoa.

Religiosity is also a foundational part of American Samoan culture and social norms. Religious leaders are highly respected and often serve as sources of emotional guidance [1], consistent with findings among Polynesian communities in other settings [27,28]. Studies among Western youth have shown that positive religious coping, which is grounded in spiritual support and the concept of God as a partner who provides guidance in times of stress in conjunction with active coping [29], is associated with positive affect and life satisfaction, while negative religious coping, which is grounded in feelings of abandonment by God and interprets life challenges as punishments from God [29], is associated with negative affect and further psychological distress [28,30]. These findings are aligned with the current study, where the utility of religion as a coping strategy among youth in American Samoa was perceived to be mixed depending on how religion was used in conjunction with other coping strategies.

The mixed category warrants further interpretation and represents an important contribution of this work. Prayer and social media were categorized as mixed not because they were the only strategies whose function could vary, but because participants explicitly described both approach-oriented and avoidance-oriented uses of these strategies. In principle, many strategies, including music, sports, sleep, community activities, or service, could support emotion regulation, connection, identity, belonging, or agency even when they do not address the underlying stressor, but could also function as avoidance depending on how they are used. Prayer was characterized as approach-oriented when it provided comfort, hope, spiritual connection, or a pathway to trusted support, but as avoidance-oriented when it was treated as a substitute for talking about distress or seeking formal mental health care. Similarly, social media was characterized as approach-oriented when it enabled expression, connection, or support-seeking, but as avoidance-oriented or potentially harmful when it encouraged comparison, concealment, external validation, exposure to judgment, or non-response to disclosures of distress. Together these findings suggest that the function of a coping strategy cannot be inferred from the behavior alone; it depends on intention, relational context, perceived safety, and the response of others.

This study has several strengths and limitations. Notably, the inclusion of *N =* 63 Samoan voices in our sample is considered rigorous [31,32] and allowed this work to reach saturation on the identified themes. The two-step incremental qualitative approach allowed us to capture and refine contextual understanding and increase the validity of identified themes. Overall, the diversity among included Samoan adult and adolescent perspectives and the involvement of Samoan voices in the design, data collection, analysis, and interpretation of results, as well as the validation of many adult perspectives among youth focus groups, strengthens the validity and reliability of this work. The culturally specific contribution of this study lies not in showing that adolescents use both approach- and avoidance-oriented coping strategies, but in illustrating how the function of these strategies is shaped by the social organization of American Samoa. This emphasis on relational, cultural, and community context is consistent with broader Indigenous and Pacific youth mental health literature, including work with Native Hawaiian youth and rural or remote Indigenous communities, which highlights cultural identity, community connection, cultural teachings, and culturally grounded support as important dimensions of wellbeing [33–35]. These findings extend prior work among Polynesian communities by showing how collectivist coping, religious support, peer connection, and stigma intersect in the everyday coping strategies available to American Samoan adolescents.

As a potential limitation of this work, many of the adult key informants worked in the mental health field in some capacity. This strengthened our ability to identify visible patterns of distress, service barriers, and existing programs, but it may also have shaped the findings toward coping strategies that are more visible to adults and professionals. Less visible, private, or peer-specific coping practices may have been underrepresented, although the adolescent focus groups helped validate and extend the adult accounts. In line with this, some findings, particularly those related to visible high-risk behaviors and service use, were identified primarily through adult key informant perspectives and should be interpreted as community and professional perceptions rather than lived adolescent accounts. With interviews conducted in English and over Zoom, there is a possibility that those with less English proficiency and challenges with connectivity were underrepresented. We believe underrepresentation based on language is unlikely, however, since English is spoken by >90% of the American Samoan population and used broadly in clinical practice and educational settings. As this is a qualitative study, the findings are limited in their generalizability, although they may be a useful starting point for work among the Samoan diaspora. Data collection occurred during and shortly after the COVID-19 pandemic, a period that disrupted schooling, social interaction, church and youth activities, and access to support. Although COVID-19 was not the central focus of interviews or focus groups, and participants did not consistently identify COVID-19 as a direct driver of the coping strategies they described, the broader pandemic context may have shaped both the coping strategies available to adolescents and the strategies most salient to participants. For example, restrictions on in-person schooling, youth groups, sports, and church gatherings may have limited access to relational and community-based coping resources, while increasing reliance on online communication and social media. As such, the findings depended on the coping strategies key informants were familiar with or had experienced first-hand, making it difficult to accurately distinguish the most common and least common coping strategies used among youth; however, given that this work was qualitative in nature, assessing the prevalence estimates of certain coping strategies over others was not an objective in this work. The interview guide was phrased in such a way that the coping strategies suggested by KIs were in response to generalized stress, and it is not possible to interpret identified coping strategies according to the contexts in which they are used. For example, we were not able to categorize coping strategies based on whether youth used them to cope with family stressors versus academic stressors. Future studies incorporating both qualitative and quantitative methods could analyze stressors and coping strategies specific to different socioecological situations, including home and family, faith, school, and community contexts. Future work could also focus on the development of a Polynesian-specific framework to describe coping strategies.

## Conclusion

Utilizing a collaborative and community-partnered approach, these findings provide a foundation of knowledge upon which to develop mental health promotion and psychoeducational initiatives to help adolescents better understand their own stress and build healthy coping strategies.

### Relevance for clinical practice

Our findings may be useful for clinical, school-based, faith-based and community organizations seeking to strengthen adolescent mental health in American Samoa. The most direct implication of this study is that programs should build on coping contexts that participants already identified as meaningful for youth, including peer networks, trusted adults, youth groups, creative and athletic activities, faith communities, school settings, and community service. This recommendation is consistent with broader calls for culturally responsive and community-grounded mental health supports for Pacific Islander and Indigenous youth [19,34,35]. Psychoeducational initiatives for parents, teachers, church leaders, coaches, and other trusted adults could help them recognize avoidance-oriented or high-risk responses to distress, respond to youth disclosures without shame or dismissal, and connect adolescents to appropriate support. Similarly, stress management programs for youth could help adolescents identify major sources of stress, recognize how they tend to cope, and distinguish strategies that promote expression, connection, and support from those that may conceal distress or increase risk.

More broadly, the findings suggest that community-based coping supports should be designed to strengthen existing sources of connection while expanding access to safer pathways for help-seeking. Sustained funding will be needed to ensure that youth programs, school-based supports, and community mental health promotion efforts remain accessible to adolescents at highest risk and to those with limited access to formal services. Although these findings may be relevant to Samoan communities beyond American Samoa, adaptation to other settings should be undertaken in partnership with local youth, families, faith leaders, clinicians, and community organizations.

### Do you need mental health support?

If you are struggling with your mental health, please call the + 988 Suicide and Mental Health Helpline (988 from a mainland phone) to be connected with a mental health counselor in American Samoa or call the US National Suicide Prevention Lifeline at 1-800-273-TALK (8255).

## References

[1] McCutchan-Tofaeono JB. Teu le va: We over me. A brief overview of mental health amongst Samoans in American Samoa. Int Cult Psychol Ser. 2021;:45–55. doi: 10.1007/978-3-030-87763-7_4

[2] Mew EJ, Blas V, Winschel J, Hunt L, Soliai-Lemusu S, Johansson A, et al. “There are still broken or fragmented systems”: Qualitative assessment of needs to strengthen adolescent mental health services in American Samoa. Int J Ment Health Nurs. 2024;33(1):85–92. doi: 10.1111/inm.13223 37691318 PMC10872981

[3] Mew EJ, Hunt L, Toelupe RLM, Blas V, Winschel J, Naseri J, et al. O le tagata ma lona aiga, o le tagata ma lona fa’asinomaga (Every person belongs to a family and every family belongs to a person): Development of a parenting framework for adolescent mental wellbeing in American Samoa. Child Youth Serv Rev. 2024;160:107502. doi: 10.1016/j.childyouth.2024.107502 38946713 PMC11209950

[4] Billings AG, Moos RH. The role of coping responses and social resources in attenuating the stress of life events. J Behav Med. 1981;4(2):139–57. doi: 10.1007/BF00844267 7321033

[5] Herman-Stabl MA, Stemmler M, Petersen AC. Approach and avoidant coping: Implications for adolescent mental health. J Youth Adolescence. 1995;24(6):649–65. doi: 10.1007/bf01536949

[6] Roth S, Cohen LJ. Approach, avoidance, and coping with stress. Am Psychol. 1986;41(7):813–9. doi: 10.1037//0003-066x.41.7.813 3740641

[7] Clausen BK, Porro D, Zvolensky MJ, Capron DW, Buitron V, Albanese BJ. Unique relations of avoidant, emotion, and problem focused coping and suicidality in a sample of sexual and gender minorities. J Affect Disord. 2025;379:473–80. doi: 10.1016/j.jad.2025.03.077 40088984

[8] Nielsen E, Sayal K, Townsend E. Exploring the Relationship between Experiential Avoidance, Coping Functions and the Recency and Frequency of Self-Harm. PLoS One. 2016;11(7):e0159854. doi: 10.1371/journal.pone.0159854 27442036 PMC4956262

[9] Wagner EF, Myers MG, McIninch JL. Stress-coping and temptation-coping as predictors of adolescent substance use. Addict Behav. 1999;24(6):769–79. doi: 10.1016/s0306-4603(99)00058-1 10628511

[10] Richardson CE, Magson NR, Fardouly J, Oar EL, Forbes MK, Johnco CJ, et al. Longitudinal Associations between Coping Strategies and Psychopathology in Pre-adolescence. J Youth Adolesc. 2021;50(6):1189–204. doi: 10.1007/s10964-020-01330-x 33118093

[11] Albqoor MA, Hamdan KM, Shaheen AM, Albqour H, Banhidarah N, Amre HM. Coping among adolescents: differences and interaction effects of gender, age, and supportive social relationships in Arab culture. Curr Psychol. 2021. doi: 10.1007/s12144-021-02179-4

[12] Darabos K, Mazza MC, Somers J, Song AV, Hoyt MA. Peer victimization and relationships to approach and avoidance coping to health and health behaviors. Behav Med. 2023;49(1):15–28. doi: 10.1080/08964289.2021.1946468 34288828 PMC8776890

[13] Cherewick M, Kohli A, Remy MM, Murhula CM, Kurhorhwa AKB, Mirindi AB, et al. Coping among trauma-affected youth: a qualitative study. Confl Health. 2015;9:35. doi: 10.1186/s13031-015-0062-5 26579210 PMC4647601

[14] Markus HR, Kitayama S. Culture and the self: implications for cognition, emotion, and motivation. Psychol Rev. 1991;98:224–53.

[15] Frías MT, Shaver PR, Díaz-Loving R. Individualism and collectivism as moderators of the association between attachment insecurities, coping, and social support. J Soc Pers Relat. 2014;31(1):3–31. doi: 10.1177/0265407513484631

[16] Allen GEK, Kim BSK, Smith TB, Hafoka O. Counseling attitudes and stigma among Polynesian Americans. Couns Psychol. 2015;44(1):6–27. doi: 10.1177/0011000015618762

[17] Allen GEK, Heppner PP. Religiosity, coping, and psychological well-being among Latter-Day Saint Polynesians in the U.S. Asian Am J Psychol. 2011;2(1):13–24. doi: 10.1037/a0023266

[18] Allen GEK, Smith TB. Collectivistic coping strategies for distress among Polynesian Americans. Psychol Serv. 2015;12(3):322–9. doi: 10.1037/ser0000039 26053646

[19] Garrett MF, Cutrer-Párraga EA, Allen GEK, Young EL, Urbina KJ, Hull IM. “It Would Ruin My Life”: Pacific Islander Male Adolescents’ Perceptions of Mental Health Help-Seeking-An Interpretative Phenomenological Analysis Focus Group Study. Int J Environ Res Public Health. 2025;22(1):62. doi: 10.3390/ijerph22010062 39857515 PMC11764690

[20] Toluono A, Deans E, Ravulo J, Conroy E. A qualitative study exploring the help seeking attitudes and barriers to support for Pasifika young people. Public Health Pract (Oxf). 2026;11:100753. doi: 10.1016/j.puhip.2026.100753 41853088 PMC12992069

[21] Blas V, Mew EJ, Winschel J, Hunt L, Soliai Lemusu S, Lowe SR, et al. Community perspectives on adolescent mental health stigma in American Samoa. PLOS Ment Health. 2024;1(5):e0000080. doi: 10.1371/journal.pmen.0000080 41661749 PMC12798432

[22] Goodyear-Smith F, ‘Ofanoa M. Fa’afaletui: A Pacific Research Framework. J Mix Methods Res. 2021;16(1):34–46. doi: 10.1177/1558689820985948

[23] Tamasese K, Peteru C, Waldegrave C, Bush A. Ole Taeao Afua, the new morning: a qualitative investigation into Samoan perspectives on mental health and culturally appropriate services. Aust N Z J Psychiatry. 2005;39(4):300–9. doi: 10.1080/j.1440-1614.2005.01572.x 15777368

[24] Braun V, Clarke V. Using thematic analysis in psychology. Qual Res Psychol. 2006;3:77–101. doi: 10.1191/1478088706qp063oa

[25] Capstick S, Norris P, Sopoaga F, Tobata W. Relationships between health and culture in Polynesia - a review. Soc Sci Med. 2009;68(7):1341–8. doi: 10.1016/j.socscimed.2009.01.002 19195751

[26] Mulipola L, Wiles J, Fa’alau F. Understanding anger with New Zealand-born Samoan youth: A Samoan qualitative exploration. Aust N Z J Public Health. 2024;48(4):100162. doi: 10.1016/j.anzjph.2024.100162 38945053

[27] Mulipola TI, Holroyd E, Vaka S. Using Fa’afaletui to explore Samoan consumers’ experience and interpretation of mental health person-centred care in Aotearoa, New Zealand. Int J Ment Health Nurs. 2023;32(2):513–23. doi: 10.1111/inm.13090 36373845

[28] Yamada A-M, Vaivao DES, Subica AM. Addressing Mental Health Challenges of Samoan Americans in Southern California: Perspectives of Samoan Community Providers. Asian Am J Psychol. 2019;10(3):227–38. doi: 10.1037/aap0000140 33777325 PMC7992925

[29] Krok D. The mediating role of coping in the relationships between religiousness and mental health. Arch Psych Psych. 2014;16(2):5–13. doi: 10.12740/app/26313

[30] Van Dyke CJ, Glenwick DS, Cecero JJ, Kim S-K. The relationship of religious coping and spirituality to adjustment and psychological distress in urban early adolescents. Mental Health Relig Cult. 2009;12(4):369–83. doi: 10.1080/13674670902737723

[31] Dworkin SL. Sample size policy for qualitative studies using in-depth interviews. Arch Sex Behav. 2012;41(6):1319–20. doi: 10.1007/s10508-012-0016-6 22968493

[32] Guest G, Bunce A, Johnson L. How many interviews are enough? An experiment with data saturation and variability. Field Methods. 2006;18(1):59–82. doi: 10.1177/1525822X05279903

[33] Riley L, Suʻesuʻe A, Hulama K, Neumann SK, Chung-Do J. Ke ala i ka Mauliola: Native Hawaiian Youth Experiences with Historical Trauma. Int J Environ Res Public Health. 2022;19(19):12564. doi: 10.3390/ijerph191912564 36231865 PMC9566730

[34] Venugopal J, Morton Ninomiya ME, Green NT, Peach L, Linklater R, George P, Wells S. A scoping review of evaluated Indigenous community-based mental wellness initiatives. Rural and Remote Health 2021;21:6203. doi: 10.22605/RRH6203 33730509

[35] Okpalauwaekwe U, Ballantyne C, Tunison S, Ramsden VR. Enhancing health and wellness by, for and with Indigenous youth in Canada: a scoping review. BMC Public Health. 2022;22(1):1630. doi: 10.1186/s12889-022-14047-2 36038858 PMC9422134

